# A Simple Grinding Method for Preparing Ultra-Thin Boron Nanosheets

**DOI:** 10.3390/nano12111784

**Published:** 2022-05-24

**Authors:** Haoran Wang, Zuxin Sun, Zuoshan Wei, Yuying Wu

**Affiliations:** 1 Key Laboratory of Liquid-Solid Structure Evolution and Processing of Materials, Ministry of Education, Shandong University, Jinan 250061, China; whr15706442929@163.com (H.W.); sunnyzx199207@gmail.com (Z.S.); 2 Shandong Key Laboratory of Advanced Aluminum Materials and Technology, Binzhou Institute of Technology, Binzhou 256600, China; 15376251006@163.com

**Keywords:** boron, nanosheet, microstructure, nanocrystalline materials

## Abstract

The preparation of boron nanosheets has very strict requirements of the preparation environment and substrate. In this work, the boron nanosheets were tried to prepare by the grinding method, using β-B alloy with stable chemical properties and large crystal plane spacing. Its morphology and chemical bonds of boron nanosheets were analyzed by scanning microscope (SEM), transmission microscope (TEM), and X-ray photoelectron spectroscopy (XPS). The results show that the two-dimensional boron nanosheets can be prepared from β-B powder by the grinding method. There are very few B-O bonds in boron particles, and the B-B bonds are principally dominant. In addition to a few B-O bonds, including some B-B bonds change to B_6_O bonds which are not completely oxidized, indicating that boron has certain oxidation resistance.

## 1. Introduction

Since boron nanosheets [1] have good chemical and physical properties, they can be used in medicine, aerospace, nuclear energy, electronic transmission, and other fields. At present, due to the easy oxidation of boron nanosheets and the high cost of the bottom-up preparation method, it needs very harsh synthesis conditions, ultra-high vacuum degrees, and sensitive temperature change. Graphene has the corresponding bulk phase layered structure in nature, and its monolayer structure can be easily analyzed. Geim [2] successfully prepared the monolayer graphene in 2004 by using a simple mechanical stripping method. However, boron has strong covalent bonds both in and out of the plane, and, thus, it is impossible to obtain monolayer structures directly through mechanical stripping.

As early as 1997, Boustani [3] calculated by the first principles that the pentagram and hexagram composed of boron atoms could be used as the basic units to form quasiplanar structures, and it was the first theoretical prediction that the monolith boron could stably exist. Subsequently, the Lau research team calculated and predicted that monolayer boron nanosheets could stably exist in the form of curly triangular lattices [4]. Yakobson [5], Zhao [6] and Gao [7] et al. calculated results showing that the monolayer boron nanosheets can be grown from MgB_2_ on the Ag (111) and Au (111) substrates, providing the preparation direction and theoretical support for experimental preparation of borylene [8]. Mannix [9] prepared boron nanosheets with an atomic thickness on the Ag surface under ultra-high vacuum conditions. Xu [10] explored the porous monolith of few-layered boron nitride for effective water cleanup. Feng [11] used molecular beam epitaxy (MBE) to grow boron sheets on Ag (111) surface under ultra-high vacuum (UHV) conditions and successfully obtained 2D boron sheets.

In this study, boron nanosheets were prepared by grinding β-B alloy with stable chemical properties, large size, and large intercellular space. β-B is obtained by extraction of Cu-5B alloy.

## 2. Experiment and Discussion

First, the melt reaction method was used to obtain a Cu-5B alloy ingot. The ingot was cut into small pieces and put into a centrifugal tube. The Cu-5B alloy strips were obtained by using a single-roll cooling device (Melting temperature 1500 °C, rotating speed 1800 r/min). The obtained alloy strips were first corroded by nitric acid and the boron powder was obtained by vacuum drying. Then, the grinding was carried out using an agate mortar for 2 h.

**Figure 1:**
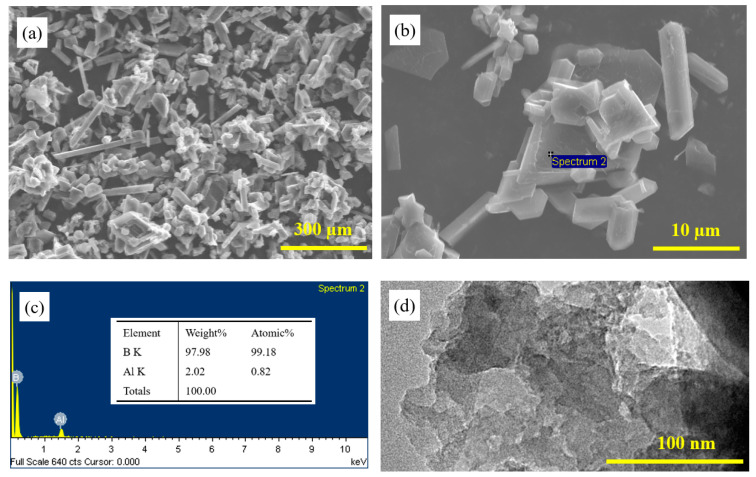

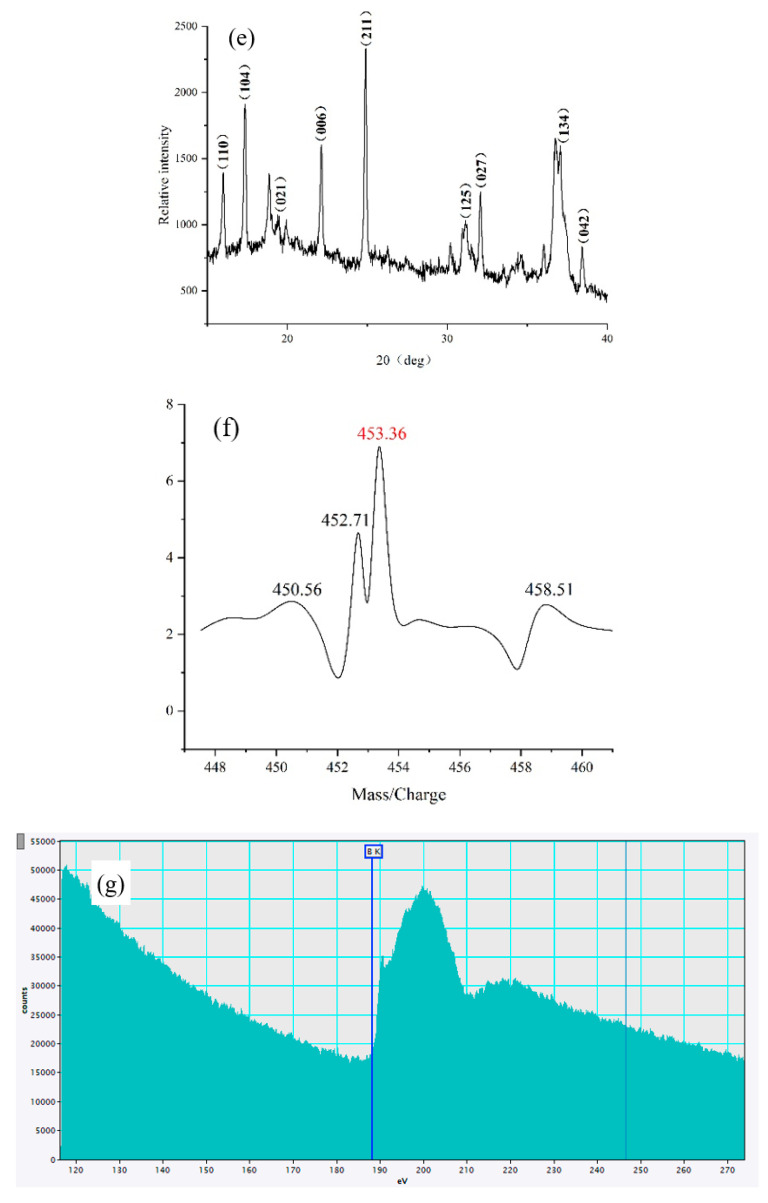
Images of boron particles and boron nanosheets before and after grinding (**a**) SEM image of original boron particles extracted from Cu-5B alloy, (**b**,**c**) EDS spectrum of boron particles, (**d**) TEM image of boron nanosheets after grinding, (**e**) XRD spectrum of boron nanosheets after grinding, (**f**) MALDI-TOF-MS result for the extracted boron powder, (**g**) the EELS analysis of boron element in boron nanosheets.

Figure 1 shows the images of boron powder before and after grinding. It could be seen that boron mainly exists as some rod-shaped and massive boron particles (Figure 1a). Figure 1b,c show EDS analysis of boron particles, which shows a boron atomic ratio of 99%, so it can be considered that the boron particles before grinding are relatively pure boron. As can be seen in Figure 1d, after grinding, some boron particles in the boron powder changed from block to sheets, and the thickness of the boron nanosheets reached nanometer level. Compared with the microcrystals before grinding, the thickness of boron nanosheets is decreased significantly, indicating that this method can be used to prepare the boron nanosheets. As shown in Figure 1e XRD spectrum of boron nanosheets after grinding. The XRD pattern of the sample is shown in the figure, with black brackets marking the crystal face of β-B. The lattice constants of β -b are as follows: A = B = 10.952 Å, C = 23.824 Å (according to PDF85-0409) [12]. MALDI-TOF-MS is used to detect the stable unit of boron. The result in Figure 1f shows that the relative atomic mass of 453.36 possesses the highest relative intensity, corresponding to B42 (B: 10.81 1) [13]. As can be seen in Figure 1g, the EELS analysis of boron element in boron nanosheets, it can be seen that boron has a very high peak strength at 200 eV.

**Figure 2:**
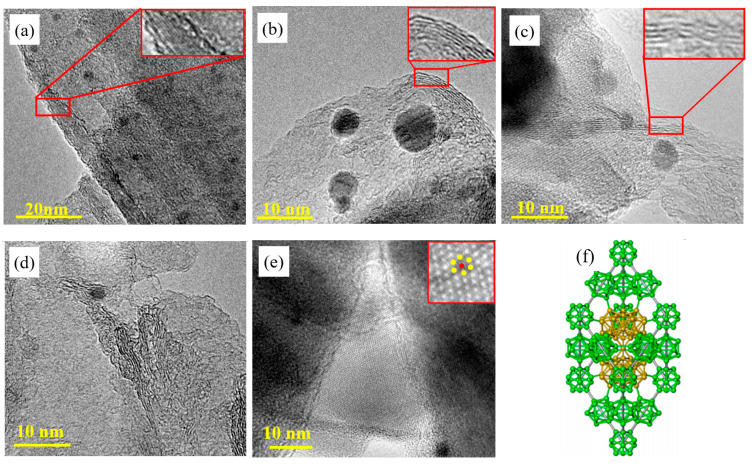
TEM images of boron nanosheets and their peeling processes (**a**) Overlapping boron nanosheets, (**b**) 5-layers boron nanosheets, (**c**–**e**) 2–3 layers of boron nanosheets, and their enlarged peeling processes, (**f**) Structure model of β-B.

**Figure 3:**
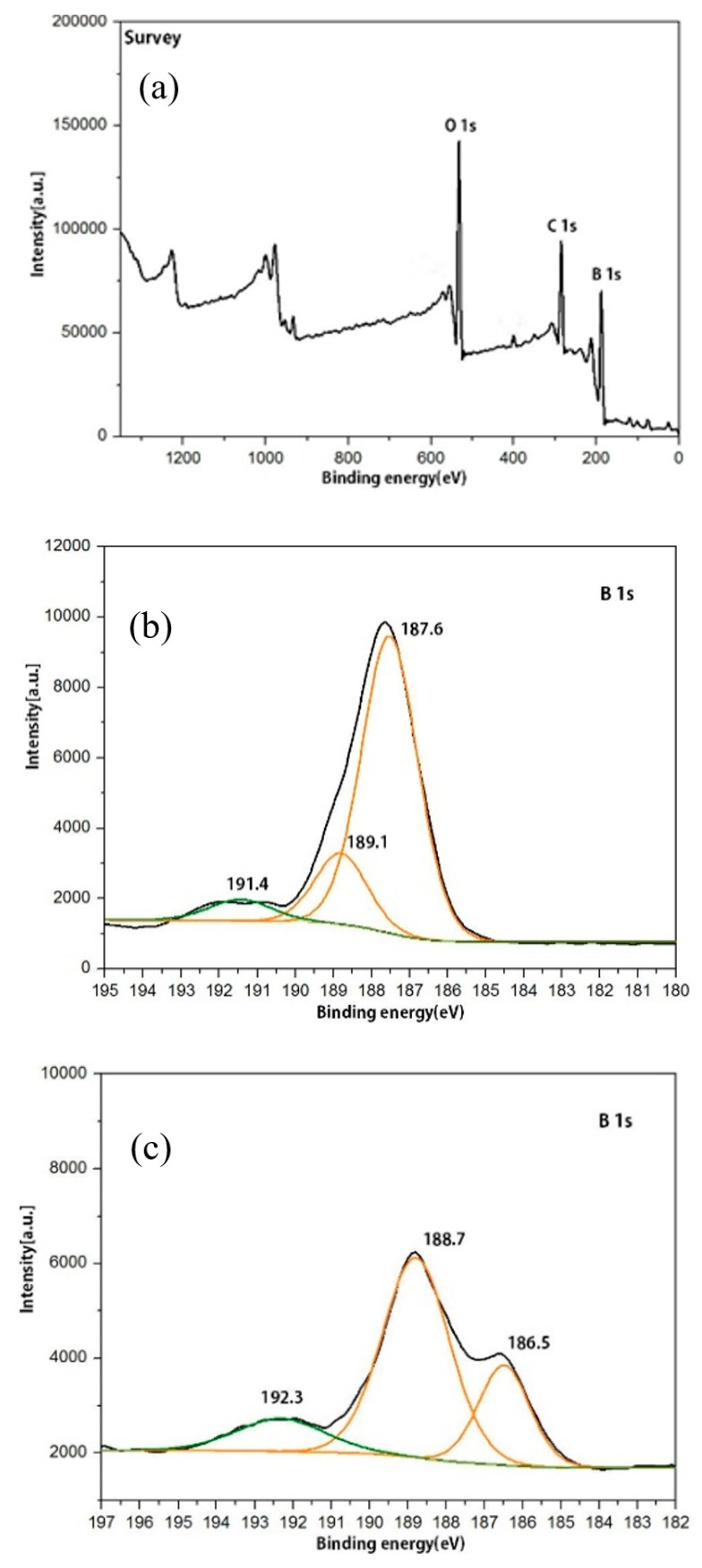
XPS results of boron powder before and after grinding (**a**) Full-scale survey (**b**) Before grinding, breakdown drawing of B 1s peak (**c**) After grinding, breakdown drawing of B 1s peak.

As can be seen in Figure 2a, some multiple boron nanosheets overlapped together and boron nanospheres with a size of about 5 nm were adsorbed at the same time. The extremely thin boron nanosheets are almost transparent under the electron beam, as shown in Figure 2b,c. The number of layers of the boron nanosheet can be seen by the crimped edges. Figure 3b shows 5layers of boron nanosheets with a size of about 40 nm. As can be observed from magnified Figure 2c,d, the process of stripping boron nanosheets from multiple layers to several layers and finally to double layers can be seen. It is indicated that the preparation of boron nanosheets by this method is through the slip between layers rather than the dissociation between layers. It is the reason why the boron nanosheet size is smaller than that of the original boron particles, the average size is about 30 nm. Figure 2e is the lattice fringe image of the boron nanosheet, with the magnified area marked by the circle. It can be measured that the exposed crystal face of the boron nanosheet is (006), and, accordingly, it can be inferred that β-B fractured along the C-axis in the grinding process. Corresponding to the crystal structure model of β-B in Figure 2f, the fracture occurred between two B12 icosahedrons (shown in green polyhedrons). β-B has a B12 icosahedron at both the vertex and the center of the edge of a single cell, and two B28 polyhedra (yellow polyhedra part in the figure) are triple fused in the middle of the cell, connected by a gap boron atom. Based on the structural model of β-B, it is believed that β-B stripping perpendicular to the C-axis may be caused by the repulsion between the B12 icosahedrons and the attraction between B12 and B28, resulting in the rupture of covalent bond between B12 units. To further characterize whether boron was contaminated by oxidation during the grinding process and the change of boron chemical environment before and after the grinding process, XPS analysis was performed.

Figure 3a is the full XPS spectrum of boron powder. Strong B1s, C1s, and O1s [14] core levels are observed in the spectrum. We fix the C1s peak at 284.8 eV as the reference to decompose the B 1s band before and after grinding, as shown in Figure 3b,c. Before grinding, the B 1s band had three peaks, indicating the existence of three different chemical environments for boron. The binding energy of block B 1s is about 189–190 eV [15]. For boron powder, the binding energy value shifts to lower energy. These two low binding energy peaks (189.1 eV and 187.6 eV) are the red-shifted relative to the location of the block boron, which is most likely the existence of two different B-B bonds in the block boron. The peak observed at 191.4 eV is attributed to the complete oxidation state of boron in B_2_O_3_, and the binding energy value is consistent with previous reports [16]. At the same time, it is observed that the proportion of boron oxide peak is very small, and mainly B-B peak with the binding energy of 187.6 eV related to B-B bonding is the most dominant.

The decomposition of B1s peak after grinding is shown in Figure 3c [17]. There were also three peaks. It can be seen that the two low binding energy peaks shift further to lower binding energies [18]. The B-B bond at the lowest binding energy (187.6 eV) before grinding is dominant, and the lower binding energy (188.7 eV) after grinding is dominant, indicating that the grinding process changes the state of two different B-B bonds. It was reported in the literature [9] that B_6_O has two different combined boron states, whose binding energies are 185.4 and 187.2 eV, respectively. It is speculated that incomplete oxidation of boron nanosheets obtained after grinding is highly likely to occur, resulting in the bonding mode of B_6_O. Thus, the lowest binding energy peak corresponds to B_6_O, the lower binding energy peak corresponds to the B-B bonds before grinding, and the high binding energy peak still corresponds to the fully oxidized B_2_O_3_. At the same time, compared with before grinding, the proportion of fully-oxidized B_2_O_3_ does not increase, indicating that boron has a certain antioxidation ability.

In conclusion, there are few B_2_O_3_ bonding and B-B bonding in two different chemical environments in boron particles before grinding. After grinding, only a few B_2_O_3_ bonds exist in the boron nanosheets, and the state of some B-B bonds changes, forming the incomplete-oxidized B_6_O bonds, but B-B bonds still account for most of them.

## 3. Conclusions

Two-dimensional boron nanosheets were prepared from β-B powder by grinding. The thickness of the boron nanosheets is different, and their size is about 40 nm. Through the analysis of XPS bonding and the chemical environment of boron, there are very few B-O bonds in boron particles, and the B-B bonds are principally dominant. In addition to a few B_2_O_3_ bonds, some B-B bonds change to B_6_O bonds, related to incomplete oxidation, indicating that boron has certain oxidation resistance.

## Data Availability

Not applicable.
